# Combined Methylome and Transcriptome Analyses Reveals Potential Therapeutic Targets for EGFR Wild Type Lung Cancers with Low PD-L1 Expression

**DOI:** 10.3390/cancers12092496

**Published:** 2020-09-03

**Authors:** Weilei Hu, Guosheng Wang, Lonny B. Yarmus, Yuan Wan

**Affiliations:** 1 Institute of Translational Medicine, Zhejiang University, Hangzhou 310029, China; weileihu@zju.edu.cn; 2 Center for Disease Prevention Research and Department of Pharmacology & Toxicology, Medical College of Wisconsin, Milwaukee, WI 53226, USA; 3 The Pq Laboratory of Micro/Nano BiomeDx, Department of Biomedical Engineering, Binghamton University—SUNY, Binghamton, NY 13902, USA; gswang@binghamton.edu; 4 Department of Medicine, Division of Pulmonary and Critical Care, Johns Hopkins School of Medicine, Baltimore, MD 21218, USA; lyarmus@jhmi.edu

**Keywords:** DNA methylation, EGFR mutation-negative, low PD-L1 expression, immune checkpoint inhibitors, combination strategies

## Abstract

**Simple Summary:**

Low expression of programmed death-ligand 1 (PD-L1), epidermal growth factor receptor (EGFR) wild-type non-small cell lung cancer (NSCLCs) are refractory, and only few therapeutic options exist. This study aims to clarify the molecular basis of this special subtype of NSCLC and identify potential therapeutic targets. We performed integrating data from multiple sources including transcriptome, methylome, and clinical outcome to uncover the effect of epigenetic changes acting this special subtype lung cancer. We elucidated both aberrant methylation and associated aberrant gene expression and the emerging methylation-transcription patterns were classified as HypoUp, HypoDown, HyperUp, or HyperDown. We found that the aberrant methylation-transcription patterns significantly affect the overall survival time of the patients. We used protein–drug interaction data and molecular docking analysis to identify potential therapeutic candidates. This study uncovered the distinct methylation-transcription characteristics of this special subtype lung cancer, and provided an adaptable way to identify potential therapeutic targets.

**Abstract:**

Immune checkpoint inhibitors (ICIs) targeting PD-1/PD-L1 have demonstrated remarkable treatment efficacy in advanced non-small cell lung cancer (NSCLC). However, low expression of programmed death-ligand 1 (PD-L1), epidermal growth factor receptor (EGFR) wild-type NSCLCs are refractory, and only few therapeutic options exist. Currently, combination therapy with ICIs is frequently used in order to enhance the treatment response rates. Yet, this regimen is still associated with poor treatment outcome. Therefore, identification of potential therapeutic targets for this subgroup of NSCLC is strongly desired. Here, we report the distinct methylation signatures of this special subgroup. Moreover, several druggable targets and relevant drugs for targeted therapy were incidentally identified. We found hypermethylated differentially methylated regions (DMRs) in three regions (TSS200, TSS1500, and gene body) are significantly higher than hypomethylated ones. Downregulated methylated genes were found to be involved in negative regulation of immune response and T cell-mediated immunity. Moreover, expression of four methylated genes (PLCXD3 (Phosphatidylinositol-Specific Phospholipase C, X Domain Containing 3), BAIAP2L2 (BAR/IMD Domain Containing Adaptor Protein 2 Like 2), NPR3 (Natriuretic Peptide Receptor 3), SNX10 (Sorting Nexin 10)) can influence patients’ prognosis. Subsequently, based on DrugBank data, NetworkAnalyst 3.0 was used for protein–drug interaction analysis of up-regulated differentially methylated genes. Protein products of nine genes were identified as potential druggable targets, of which the tumorigenic potential of XDH (Xanthine Dehydrogenase), ATIC (5-Aminoimidazole-4-Carboxamide Ribonucleotide Formyltransferase/IMP Cyclohydrolase), CA9 (Carbonic Anhydrase 9), SLC7A11 (Solute Carrier Family 7 Member 11), and GAPDH (Glyceraldehyde-3-Phosphate Dehydrogenase) have been demonstrated in previous studies. Next, molecular docking and molecular dynamics simulation were performed to verify the structural basis of the therapeutic targets. It is noteworthy that the identified pemetrexed targeting ATIC has been recently approved for first-line use in combination with anti-PD1 inhibitors against lung cancer, irrespective of PD-L1 expression. In future work, a pivotal clinical study will be initiated to further validate our findings.

## 1. Background

Although tyrosine kinase inhibitors (TKI) have shown remarkable benefits against lung cancer, they are not effective for epidermal growth factor receptor (EGFR) mutation-negative patients. More recently, the introduced immune checkpoint inhibitors (ICIs) therapy has shown marked clinical responses, especially effective towards these cases [1,2,3,4]. In the KEYNOTE 024 phase III trial, pembrolizumab, an anti-programmed death 1 (PD1) antibody, showed better therapeutic effect than standard chemotherapy against EGFR wild type lung cancers overexpressing programmed death-ligand 1 (PD-L1) [5]. In addition to findings of the KEYNOTE 024 trial, results of the KEYNOTE 42 trial [6] which included any PD-L1 positive non-small cell lung cancer (NSCLC) patients led to the approval of pembrolizumab as the first-line single agent for the treatment of metastatic NSCLC. Indeed, immunotherapy is the first-line treatment of advanced stage NSCLC patients harboring EGFR/ALK (ALK receptor tyrosine kinase) wild type with PD-L1 expression ≥ 50%, and is the second-line treatment when PD-L1 expression ranges between 1 and 50%. However, some patients, including cases with low PD-L1 expression, often do not benefit from this treatment. Thus, regimens combining PD-1/PD-L1 blockade with other approaches, including chemotherapy, have been created with the aim of enhancing response rates. In the KEYNOTE 021 study, combining chemotherapy with pembrolizumab increased overall response rate by about 57% relative to chemotherapy alone (13%) in cases exhibiting low PD-L1 levels. However, combined treatment exhibited modest improvement on overall survival (OS) and was associated with more treatment-related adverse effects in grade 3 and 4 [7,8,9]. Other approved combination regimens involve inhibitors of vascular endothelial growth factor (VEGF) [10] and cytotoxic T-lymphocyte-associated protein 4 (CTLA-4) [11,12]. Experimental combination regimens include lymphocyte-activation gene 3 (LAG-3) [13,14] and T-cell immunoglobulin mucin 3 (TIM-3) [15,16]. However, establishing the best combination regimen for EGFR wild type lung cancers with low PD-L1 expression remains daunting. In addition, both carcinogenic mechanisms and molecular basis of this special subtype of NSCLC are still elusive.

Epigenetic modification ensures the maintenance and inheritance of gene expression programs through cell division. It includes DNA methylation, which occurs predominantly at CpG dinucleotides in mammals [17,18]. Previous studies have proven that DNA methylation readers and writers are vital components of the adaptive immune response [19,20,21,22,23]. DNA methylation is also implicated in T-cell exhaustion, and blocking epigenetic processes may promote T cell rejuvenation, thus supporting the anti-tumor effects of checkpoint blockade [24]. In NSCLC, epigenomics has been shown to influence clinical effects of anti-PD-1 therapy [25]. Aberrant DNA methylation has been shown to enhance resistance to immunotherapy in lung cancer [26]. However, few studies have investigated DNA methylation changes in EGFR wild type lung cancers with low PD-L1 expression.

In this study, based on the multiple platforms utilized within the Cancer Genome Atlas (TCGA), we performed integrating data from multiple sources including transcriptome, methylome, and clinical outcome to uncover the effect of epigenetic changes acting in the development and progression of EGFR wild type lung cancers with low PD-L1 expression. Notably, to minimize noise from unrelated methylations and gene expression, methylation sites and associated genes were treated as single units. We elucidated both aberrant methylation and associated aberrant gene expression and the emerging methylation/expression patterns were classified as HypoUp, HypoDown, HyperUp, or HyperDown. The results also indicated that aberrant methylation-transcription patterns significantly affect the overall survival time of the patients since a risk assessment model including four differentially methylated and expressed genes (DMEGs; PLCXD3 (Phosphatidylinositol-Specific Phospholipase C, X Domain Containing 3), BAIAP2L2 (BAR/IMD Domain Containing Adaptor Protein 2 Like 2), NPR3 (Natriuretic Peptide Receptor 3), SNX10 (Sorting Nexin 10)) successfully categorized patients into high- and low-risk classes. Furthermore, FDA-approved drugs targeting up-regulated differentially methylated genes were explored using protein–drug interaction data from DrugBank database. The binding mode of target-drug complex was verified through molecular docking analysis and molecular dynamics simulation at a molecular level, deserving further investigation to validate.

## 2. Materials and Methods

### 2.1. Sample Datasets and Data Preprocessing

Publicly available NSCLC and adjacent non-cancer tissue gene expression (RNA-SeqV2) and methylation data (Illumina Infinium HumanMethylation450 BeadChip; Illumina, San Diego, CA, USA), and corresponding clinical data were downloaded from TCGA on 2 March 2020. These data comprised of 108 normal samples and 133 EGFR wild type lung cancer samples with low PD-L1 expression, of which 75 normal samples and 115 above-mentioned tumor samples contained both gene expression and DNA methylation data. The mutation annotation format files of 132 tumor samples (one was missing) were also downloaded, and clinical sample characteristics are detailed in Table S1. The bottom 25% samples, with regards to PD-L1 expression, were considered PD-L1 low expression. The NSCLC expression dataset and methylation data as well as the corresponding clinical information in Gene Expression Omnibus (GEO) were included to validate our results (GSE31210).

### 2.2. Immune Profile Analysis

Tumor-infiltrating lymphocytes including B, and dendritic cells, neutrophils, CD8+ T, macrophages, CD4+ T, was analyzed among “EGFR Wild Type/Low PD-L1 expression” NSCLC and normal samples using tumor immune estimation resource (TIMER; https://cistrome.shinyapps.io/timer). The expression scores of micro-environmental factors (tumor, immune, and stromal purity) were obtained using the ESTIMATE (Estimation of STromal and Immune cells in MAlignant Tumor tissues using Expression data) algorithm [27].

### 2.3. Analysis of DNA Methylation Data

The Illumina HumanMethylation450 BeadChip array is comprised of 485,577 probes covering 99% (*n* = 21,231) of the RefSeq gene. For each probe, the raw methylation intensity was expressed as a β value [28]. Differentially methylated CpG sites (DMS) were identified using the R package limma by comparing CpG site data in normal samples relative to EGFR wild type lung cancer samples with low PD-L1 expression. *p* values were converted to false discovery rate (FDR) using the Benjamini and Hochberg (BH) method. FDR < 0.01 and absolute delta β-value > 0.2 were set as cutoff thresholds for DMS identification. CpG sites associated with genes were obtained from an annotation file provided by Illumina (https://www.illumina.com/). Average β-values of genes within different gene regions (TSS1500, TSS200, 5′-UTR, first exon, gene body, 3′-UTR, and intergenic region) were calculated based on correspondences [29]. Differentially methylated regions (DMRs) were calculated from the integrated methylation data using the R package limma using the following criteria: hypermethylated DMRs with FDR < 0.01 and delta β-value > 0.2; hypomethylated DMRs with FDR < 0.01 and delta β-values < −0.2. Differentially methylated genes (DMGs) were characterized by genes located in DMRs.

### 2.4. Gene Expression Data Analysis

Differentially expressed genes in normal vs. “EGFR Wild Type/Low PD-L1 expression” NSCLC TCGA datasets were identified using the R package limma and *p* values converted to FDR using the BH method. Differentially expressed genes (DEGs), were identified by log2 transformation of TCGA gene expression data and the following criteria: upregulated genes had FDR > 0.01 and log2FC > 1; downregulated genes had FDR > 0.01 and log2FC < −1 in tumor samples relative to non-cancer tissue.

### 2.5. Analysis of DMGs and DEGs in Different Regions

To uncover relationships between methylation and expression profiles, DMGs and DEGs intersections were analyzed to identify DMEGs. The DMEGs fell into 4 groups (Table 1).

### 2.6. Functional Enrichment Analysis

Gene Ontology (GO) term and Kyoto Encyclopedia of Genes and Genomes (KEGG) pathway analysis of DMGs, DEGs was done using the R package clusterProfiler. Gene enrichment analysis for DMEGs was carried out by Metascape (http://metascape.org), a web tool for gene annotation [30].

### 2.7. Evaluation of Expression and Methylation Biomarkers

Principal Component Analysis (PCA) of the DMSs in DMEGs was used to distinguish between tumor and non-tumor samples. The R package randomForest was used to classify samples based on DMEGs expression profiles and DMSs methylation profiles and validated using the leave-one-out cross-validation (LOOCV) approach. The results were then visualized using receiver operating characteristic (ROC) curve and area under the curve (AUC) analyses.

### 2.8. Construction of DMEGs-Based Prognostic Signature

Prognostic data were created on the expression matrix of DMEGs and matched survival data. The Least Absolute Shrinkage and Selection Operator (LASSO) regression analysis was performed for identifying DMEGs with prognostic value by R package glmnet.

### 2.9. Identification of Potential Drug Targets

NetworkAnalyst3.0 (http://www.networkanalyst.ca/), a web-based tool for analyzing and interpreting system-level gene expression data, was used to carry out protein–drug interactions analysis on the Up-expressed and Down-expressed DMEGs. Protein and drug target information was obtained from DrugBank (Version 5.0).

### 2.10. Homologous Modeling

To evaluate the binding energy and interaction patterns between drug candidate and their targets, AutodockVina 1.1.2, a silico protein–ligand docking software was employed [31]. As the absence of a complete crystal structure of SLC7A11, its theoretical structure was obtained from homology modeling by Swiss-Model server, using the crystal structure of large neutral amino acids transporter small subunit 1 (PDB ID: 6irt.1.B) as the template. Molecular dynamics simulation was carried out by GROMACS 5.0.6 [32]. Ramachandran plots were used to assess stereo-chemical quality [33]. The parameters were set to default.

### 2.11. Molecular Docking

The 3D structures of all candidate drug compounds were drawn by ChemBioDraw Ultra 17.0 and then subjected to energy optimization by the MMFF94 force field. The 3D structure of XDH (PDB ID: 2e1q), ATIC (PDB ID: 1pl0), CA9 (PDB ID: 5fl6), GAPDH (PDB ID: 3gpd) were downloaded from the PDB (http://www.rcsb.org/pdb/home/home.do), and 3D structure of SLC7A11 was obtained from homologous modeling. Before docking analysis, all protein and molecular files were converted into PDBQT format using AutodockTools 1.5.6. Molecular docking analysis were carried out by Autodock Vina 1.1.2. The docking parameter ‘exhaustiveness’ was set to ‘20’, and other parameters were set to default. The conformation with the highest score was selected to further analyze using Free Maestro 11.9. Pymol software 2.3 was applied for model visualization and MOE software 2019 was used for drawing the 2D depictions [34].

## 3. Results

### 3.1. DMGs in “EGFR Wild Type/Low PD-L1 Expression” NSCLC

**Figure 1:**
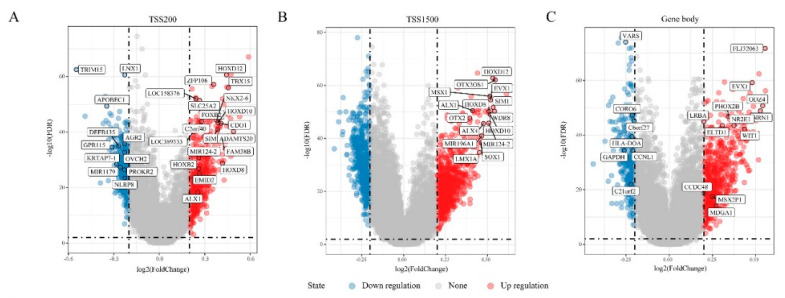

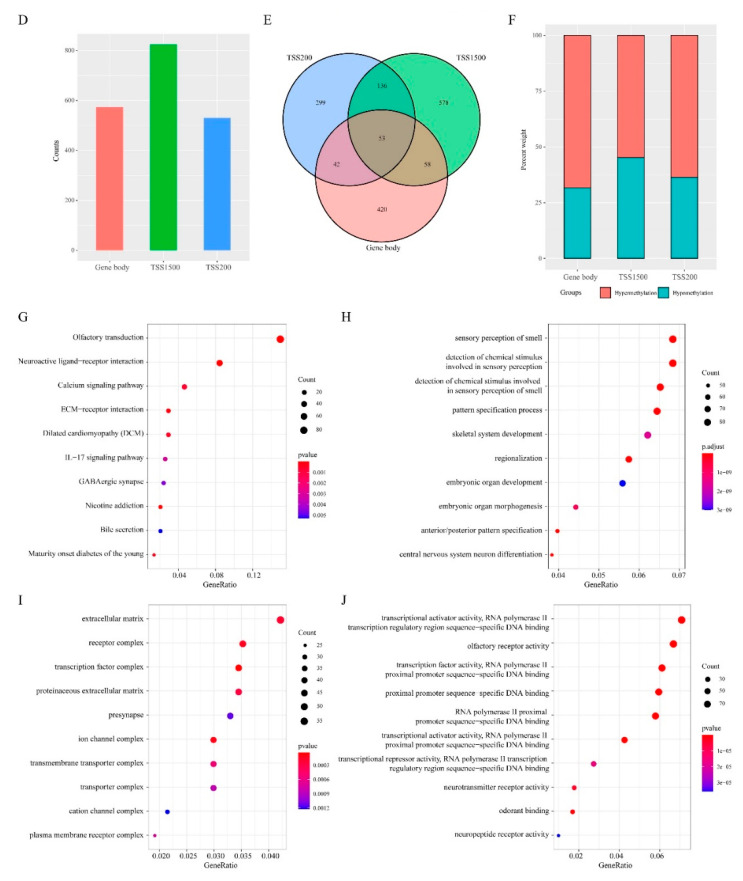
Differentially methylated genes (DMGs) in “Epidermal growth factor receptor (EGFR) Wild Type/Low PD-L1 expression” non-small cell lung cancer (NSCLC). (**A**–**C**) Volcano plots showing the distribution of DMGs in TSS200, TSS1500 and gene body regions, respectively. (**D**) Histogram showing the amount of DMGs in gene body (*n* = 573), TSS1500 (*n* = 825) and TSS200 (*n* = 530) regions. (**E**) Venn map of DMGs in three different regions. (**F**) Histogram showing the percentage of hypermethylated and hypomethylated DMGs in three different regions. (**G**) Top 10 Kyoto Encyclopedia of Genes and Genomes (KEGG) enrichment pathways of DMGs in three regions. (**H**) Top 10 Gene Ontology (GO) Biological Process (BP) terms of DMGs in three regions. (**I**) GO cellular component (CC) terms of DMGs in three regions. (**J**) GO molecular functions (MF) terms of DMGs in three regions. The size of the dots represents the number of genes enriched in the pathway, and the colors correspond to different false discovery rate (FDR) values.

To identify differential methylation in “EGFR Wild Type/Low PD-L1 expression” NSCLC, DNA methylation data from 115 tumor samples and 75 corresponding non-tumor tissues was extracted for comparative analysis. This analysis focused on the transcription start sites TSS200, TSS1500, and the gene body, and identified 3250 DMRs (FDR < 0.01, |delta β-values| > 0.2) that were annotated to 1586 genes (Figure 1A–C). The DMRs were then divided into 593 hypermethylated DMRs and 339 hypomethylated DMRs in the TSS200 region, 747 hypermethylated DMRs and 618 hypomethylated DMRs in the TSS1500 region, and 651 hypermethylated DMRs and 302 hypomethylated DMRs in gene body (Figure 1D or Figure 1F). Altogether, there were significantly more hypermethylated DMRs than hypomethylated ones. Of the 3 gene regions, TSS1500 was associated with the majority of DMGs (Figure 1E). Of the 1586 DMGs harboring DMRs, 53 genes were present in all 3 regions, 236 genes were present in at least 2 regions, and 1297 were present in one region (Figure 1E). To assess DMGs function, we performed GO functional enrichment and KEGG pathway analyses. The DMGs fell into 20 KEGG pathways (top-10 are shown on Figure 1G), while 185 were annotated to GO biological process (BP) (Figure 1H), 36 to GO term cellular component (CC) (Figure 1I), and 39 to GO term molecular functions (MF) (Figure 1J). Together, this showed that the DMGs are involved in important pathways, biological processes and cellular component, including ECM (extracellular matrix)-receptor interaction, extracellular matrix, receptor complex, transcriptional activator activity, and RNA polymerase II transcription regulatory region sequence-specific DNA binding (Figure 1G–J).

### 3.2. Immune Profile Analysis

**Figure 2:**
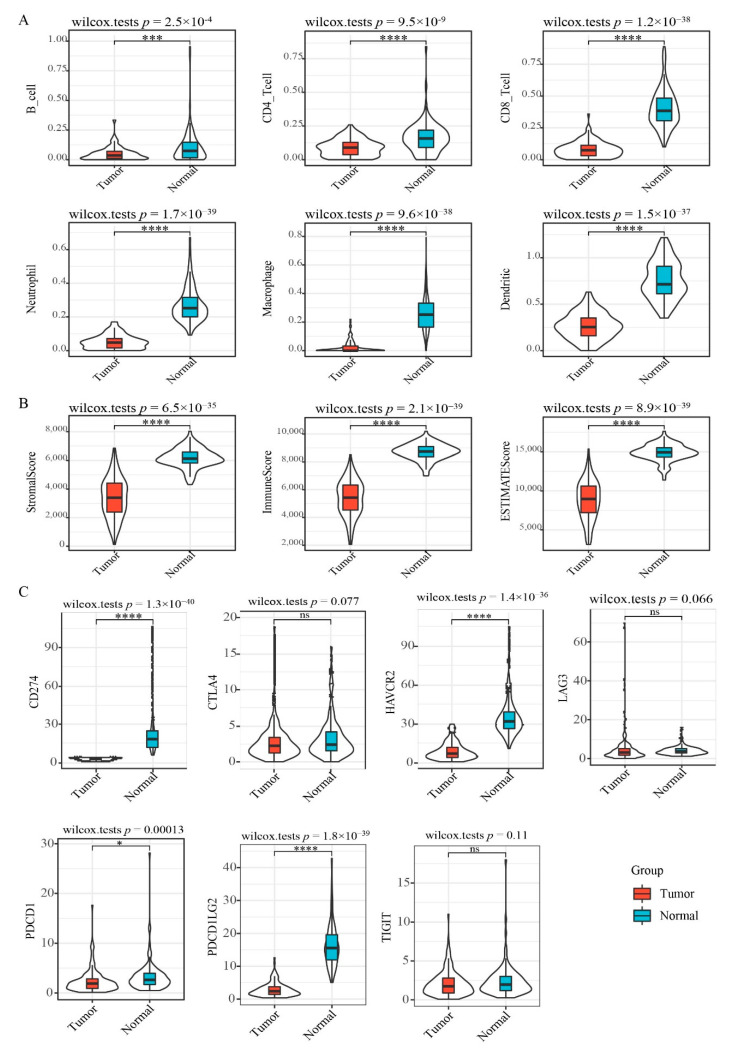
Immune signature scores in “EGFR Wild Type/Low PD-L1 expression” NSCLC. (**A**) The expression scores of immune-associated cells included in the TIMER algorithm. (**B**) The expression scores of genes included in the ESTIMATE algorithm for determination of stromal and immune gene signatures. (**C**) Differential expression of immune checkpoint molecules. Asterisks indicate significant differences (Wilcox test). * *p* < 0.05, *** *p* < 0.001, **** *p* < 0.0001, ns means no significant.

To characterize the immune cell profile of EGFR wild type lung cancer samples with low PD-L1 expression, we analyzed the expression of 6 immune cells: B cell, CD4+ T cell, CD8+ T cell, neutrophils, macrophage and dendritic cells, using TIMER and found all immune cell types to be significantly lower in “low PD-L1 expression” NSCLC relative to controls (Figure 2A), suggesting immunosuppression in double-negative NSCLC. Validation of immune status using ESTIMATE revealed that ImmuneScore, StromalScore, and ESTIMATEScore were significantly lower in “low PD-L1 expression” NSCLC samples relative to controls (Figure 2B). Assessment of immune checkpoint gene expression showed that most checkpoint genes are significantly downmodulated in “EGFR Wild Type/Low PD-L1 expression” NSCLC (Figure 2C), including CD274 (CD274 molecule; also known as PD-L1), HAVCR2 (Hepatitis A Virus Cellular Receptor 2; also known as TIM3), PDCD1 (Programmed Cell Death 1; also known as PD1), and PDCD1LG2 (Programmed Cell Death 1 Ligand 2). CTLA4 (p = 0.077) and LAG3 (p = 0.066) showed a borderline significance.

### 3.3. Differentially Expressed Genes (DEGs) in *“EGFR Wild Type/Low PD-L1 Expression*” NSCLC

**Figure 3:**
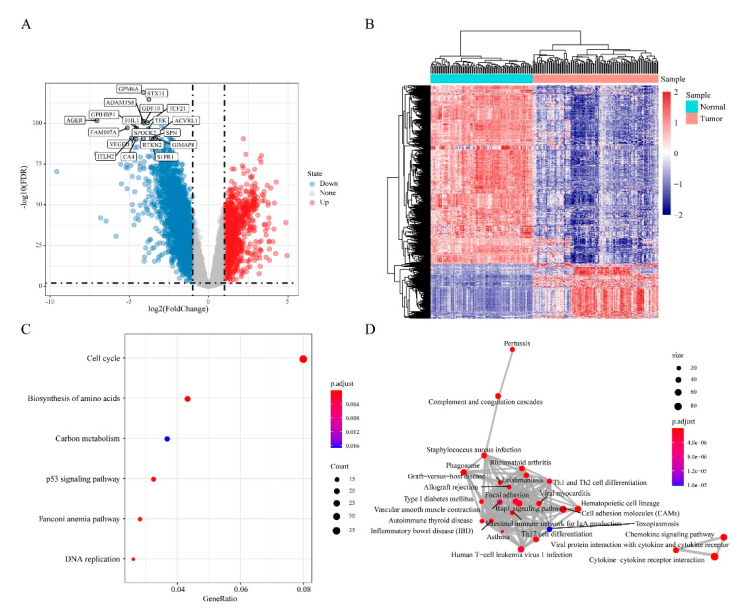
Differentially expressed genes (DEGs) in “EGFR Wild Type/Low PD-L1 expression” NSCLC. (**A**) Volcano plot showing the distribution of DEGs. (**B**) Heat map and hierarchical clustering analysis of DEG. (**C**) Significantly enriched KEGG categories show differentially up-regulated genes. (**D**) Significantly enriched KEGG categories show differentially down-regulated genes. The size of the dots represents the number of genes enriched in the pathway, and the colors correspond to different FDR values. The lines represent the intersection of genes between pathways.

To identify DEGs in “EGFR Wild Type/Low PD-L1 expression” NSCLC, gene expression data from 133 “EGFR Wild Type/Low PD-L1 expression” NSCLC samples and 108 normal samples were extracted and comparative analysis done using limma package on R. This analysis uncovered 3178 DEGs (FDR < 0.01, |log2FC| > 1). Of these, 1037 were upregulated and 2141 downregulated in “EGFR Wild Type/Low PD-L1 expression” NSCLCs (Figure 3A). Next, unsupervised hierarchical clustering analysis of the DEGs clearly distinguished “EGFR Wild Type/Low PD-L1 expression” NSCLCs samples from controls (Figure 3B). Enrichment functional analysis of DEGs using the R package ClusterProfiler revealed upregulated DEGs to be enriched in 6 functional pathways involved in NSCLC-related biological processes, including cell cycle, biosynthesis of amino acids, carbon metabolism, P53 signaling pathway, Fanconi anemia pathway, and DNA replication (Figure 3C). Downregulated DEGs were enriched in 86 pathways, mainly Th1 and Th2 cell differentiation and other pathways that are closely related to tumor development (Figure 3D).

### 3.4. Differentially Methylated and Expressed Genes (DMEGs) in “EGFR Wild Type/Low PD-L1 Expression” NSCLC

**Figure 4:**
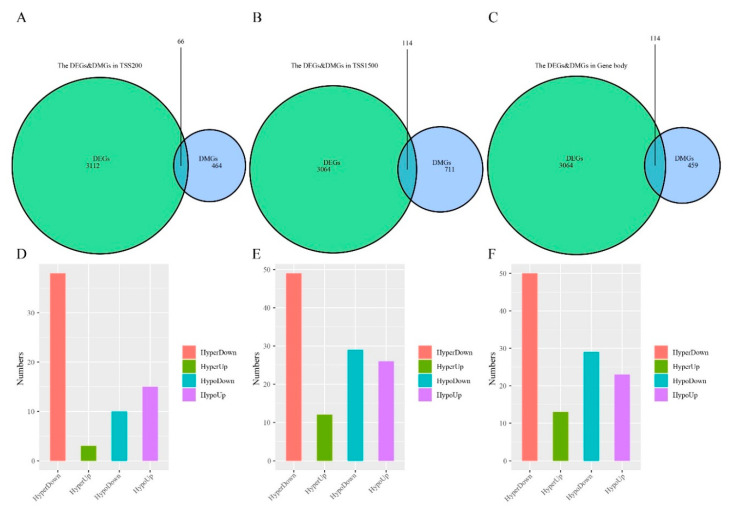
Differentially methylated and expressed genes (DMEGs) in “EGFR Wild Type/Low PD-L1 expression” NSCLC. (**A**–**C**) Venn map showing the DMEGs between DMGs and DEGs in TSS200, TSS1500, and gene body regions. (**D**–**F**) Histogram showing the number of four regulation patterns between methylation and expression of “EGFR Wild Type/Low PD-L1 expression” NSCLC in three regions.

To characterize the relationship between gene methylation and expression, we analyzed DMGs and DEGs intersection in TSS200, TSS1500, and gene body regions (Figure 4A–C). This analysis identified 249 differentially methylated and expressed genes (DMEGs) that fell into 4 classes: HypoUp (delta β-value < −0.2 and log2FC > 1), Hypodown (delta β-value > 0.2 and log2FC > 1), HyperUp (delta β-value > 0.2 and log2FC > 1) and HyperDown (delta β-value > 0.2 and log2FC < −1) (Figure 4D–F, Tables S2–S4). Of these, 209 DMEGs occurred in 1 region, 32 in 2 regions, and 8 in all 3 regions (Figure S1). The HyperDown group was most common, occupying 57.58%, 42.24% and 43.48% of the 3 regions, respectively (Figure 4D–F), followed by the HypoDown group that occupied most positions of TSS1500 and gene body regions.

### 3.5. DMEGs Analysis

**Figure 5:**
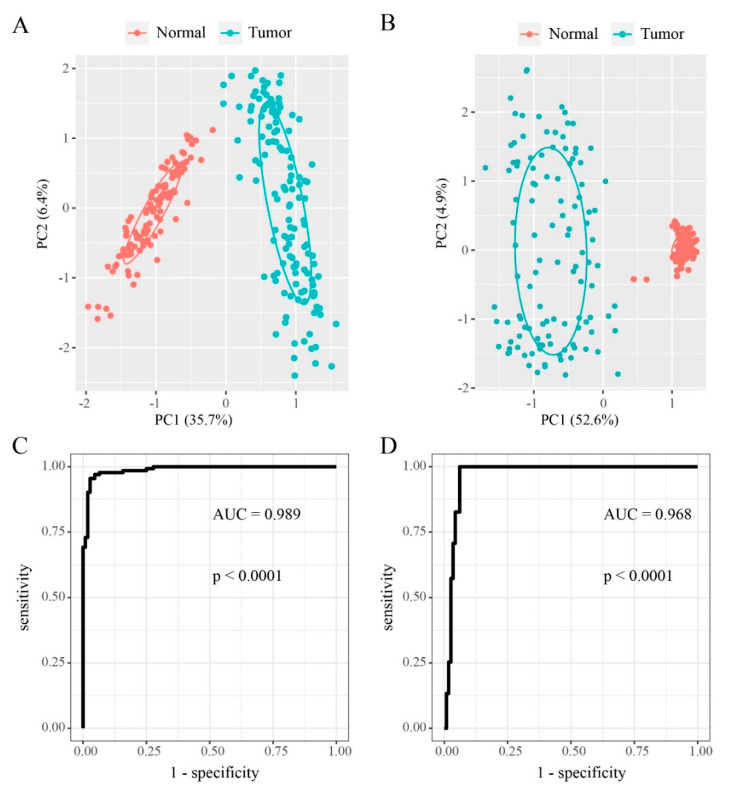
Prediction of “EGFR Wild Type/Low PD-L1 expression” NSCLC by DNA methylation and gene expression pattern. (**A**,**B**) Principal component analysis (PCA) analysis for “EGFR Wild Type/Low PD-L1 expression” NSCLC and normal samples by the 249-DMEGs and 297-DMSs predictors, respectively. (**C**,**D**) Receiver operating characteristics (ROC) displaying the classification accuracy of 249-DMEGs predictor and 297-DMSs predictors, respectively.

**Figure 6:**
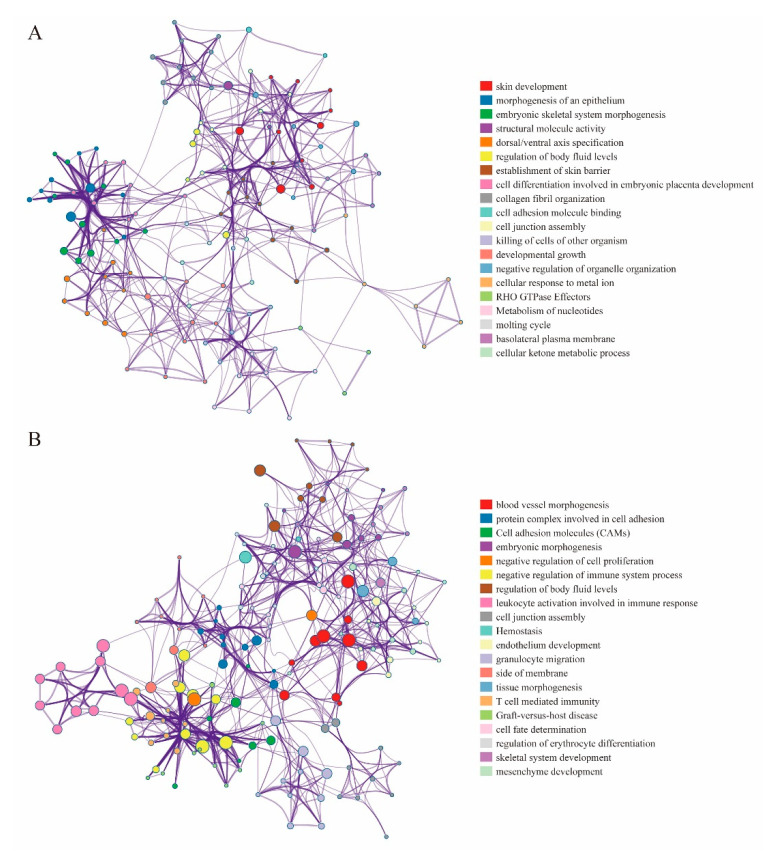
Pathway enrichment analysis of the upregulated and downregulated DMEGs. (**A**) The pathway enrichment results of upregulated DMEGs. (**B**) The pathway enrichment results of downregulated DMEGs. Each node represents an enriched term. The node size is proportional to the total number of genes in each gene set. The proportion of shared genes between genomes is indicated by the line thickness between nodes.

Our coupled analysis identified 249 DMEGs containing 297 DMSs distributed across TSS200, TSS500 and the gene body. The 297 DMSs occur throughout the genome except the sex chromosomes. To evaluate DNA methylation and gene expression differences between “EGFR Wild Type/Low PD-L1 expression” NSCLC samples and non-tumor samples, we constructed 249-DMEGs and 297-DMSs-based random forest classifiers, followed by PCA and ROC analyses. This analysis confirmed that all samples were correctly classified (Figure 5A,B). The ROC curve revealed that the 249-DMEGs classifier had an AUC value of 0.989 (p = < 0.0001, Figure 5C), while the 297-DMSs classifier had an AUC value of 0.968 (p = < 0.0001, Figure 5D). Confirming the existence of differential methylation and expression in “EGFR Wild Type/Low PD-L1 expression” NSCLC samples relative to controls. To explore the potential role of DMEGs in the occurrence and development of “EGFR Wild Type/Low PD-L1 expression” NSCLC, we divided the identified DMEGs into upregulated (78 DMEGs) and downregulated (171 DMEGs) groups. Metascape, the free gene annotation web tool, was employed to conduct pathway enrichment analysis. It was showed that upregulated DMEGs were mainly enriched in development-related signaling pathways such as skin development, morphogenesis of an epithelium, embryonic skeletal system morphogenesis, structural molecule activity, and dorsal/ventral axis specification (Figure 6A). Similarly, downregulated DMEGs were also enriched in several development-related pathways, such as blood vessel morphogenesis, embryonic morphogenesis, endothelium development, regulation of erythrocyte differentiation, and mesenchyme development (Figure 6B). It was worth noting that downregulated DMEGs were also highly enriched in immune-related pathways, including leukocyte activation involved in immune response, granulocyte migration, T cell mediated immunity, graft-versus-host disease, suggesting that downregulated DMEGs were involved in regulating immune responses of “EGFR Wild Type/Low PD-L1 expression” NSCLC, and even the formation of tumor immune microenvironment (Figure 6B). Collectively, the identified DMEGs are involved in the biological processes of the development and progression of “EGFR Wild Type/Low PD-L1 expression” NSCLC.

### 3.6. Construction and Evaluation of DMEGs-Based Prognostic Signature

**Figure 7:**
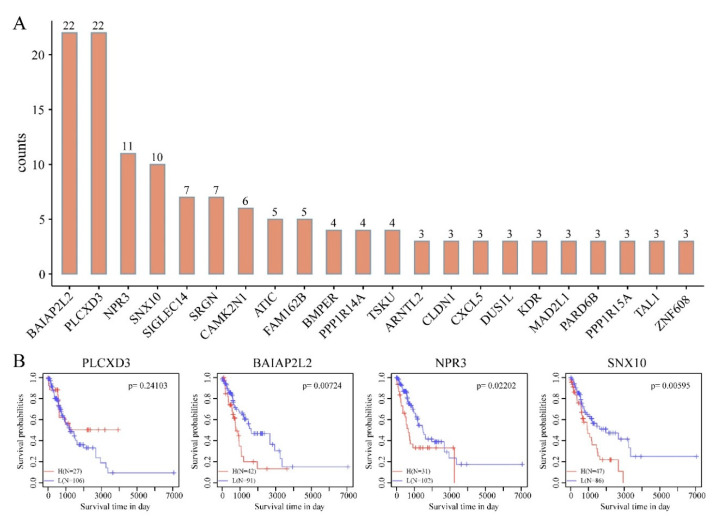
Identification of 4-DMEG risk signature for survival by Least Absolute Shrinkage and Selection Operator (LASSO) regression analysis. (**A**) Number of occurrences of each probe in 100 rounds of random sampling. (**B**) Kaplan-Meier (KM) analysis of 4 candidate DMEGs with frequencies ≥10 rounds.

To evaluate the prognostic power of the DMEGs in “EGFR Wild Type/Low PD-L1 expression” NSCLC, we constructed a DMEG-based prognostic model using LASSO regression. In this analysis of gene expression and survival data of 249 DMEGs, 200 rounds of random sampling, 80% of samples being taken each time, were performed. Next, results of each sampling were subjected to LASSO regression analysis, triple cross-validation to summarize dimensionality reduction results of each round, and counting of the number of occurrences of each probe in 100 rounds. Finally, 4 candidate DMEGs (PLCXD3, BAIAP2L2, NPR3 and SNX10), with frequencies ≥10 rounds, were selected and used to develop the prognostic model (Figure 7A,B). KM analysis revealed that all 3 genes (BAIAP2L2, NPR3, SNX10), except PLCXD3, accurately split the training set into 2 groups—high- and low-risk (Figure 7B, Table 2). The RiskScore formula used was as follows:

RiskScore_4_ = 0.022 × exp^BAIAP2L2^ + 0.011 × exp^NPR3^ − 0.102 × exp^PLCXD3^ + 0.017 × exp^SNX10^

**Figure 8:**
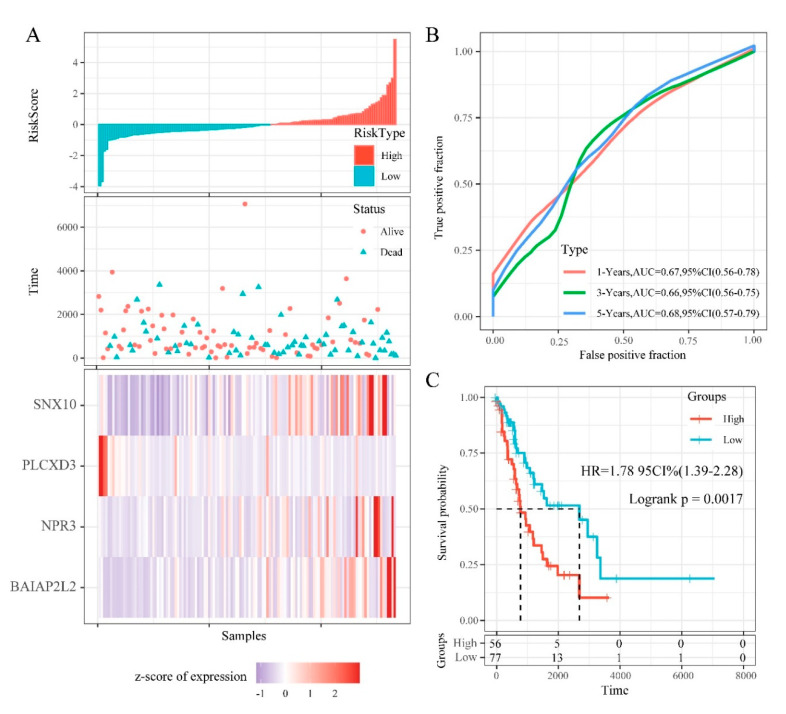
The relationship between RiskScore and patient outcome in the training cohort (from the Cancer Genome Atlas (TCGA)). (**A**) Each patient’s RiskScore, survival time, and status, and the expression of 4 DMEGs. The horizontal axis represents the samples, and the vertical axis represents RiskScores, OS (overall survival), and immune-related gene expression, respectively. (**B**) 1-, 3-, and 5-years ROC analysis of prognosis classification for RiskScore. (**C**) KM survival analysis of patients with high RiskScore vs. low RiskScore.

RiskScore distribution, survival status, and expression profile of the 4 prognostic DMEGs signatures in the training cohort are shown on Figure 8A. This analysis revealed that samples with a high RiskScore have significantly lower OS relative to those with a low RiskScore. Elevated levels of BAIAP2L2, NPR3, and SNX10, were associated with high risk, highlighting them as risk factors. While elevated PLCXD3 levels correlated with low risk, suggesting it is a protective factor. ROC analysis of RiskScore for prognostic classification, using the R package timeROC, revealed that our prognostic model has a high area under the AUC line, with the AUCs for predicting 1-, 3-, and 5-year OS being 0.67, 0.66, 0.68, respectively (Figure 8B). Finally, Zscore analysis of RiskScore was used to categorize samples with scores > 0 into the high-risk group and those with < 0 into the low-risk group. Then, 56 samples were classified into high-risk group and 77 samples into low-risk group. KM analysis revealed significant survival differences in the 2 groups (log rank *p* = 0.0017, HR = 1.78) (Figure 8C).

To assess the predictive value of this 4-DMEG-based signature, the RiskScore formula was applied to external validation set (GSE31210) and analysis was performed as in the training set. SNX10 was identified as risk factor and PLCXD3 as a protective factor (Figure S2A). AUCs for predicting 1-, 3-, and 5-year OS in the validation cohort were 0.51, 0.65, and 0.67, respectively (Figure S2B). 101 samples were classified as high-risk and 125 samples as low-risk. KM analysis revealed significant survival differences between high- and low-risk groups (log rank *p* = 0.037, HR = 1.48) (Figure S2C).

### 3.7. Multiple DMEGs Are Potential Druggable Targets

To explore whether there are any available drugs targeting DMEGs, NetworkAnalyst 3.0 was employed for protein–drug interaction analysis of up-regulated DMEGs using data from DrugBank. Protein products of 9 DMEGs were identified as drug interacting (Table 3). The majority of these, including XDH (Xanthine Dehydrogenase) [35,36], ATIC (5-Aminoimidazole-4-Carboxamide Ribonucleotide Formyltransferase/IMP Cyclohydrolase) [37], CA9 (Carbonic Anhydrase 9) [38], SLC7A11 (Solute Carrier Family 7 Member 11) [39], and GAPDH (Glyceraldehyde-3-Phosphate Dehydrogenase) [40] are implicated in tumorigenesis. XDH, which encodes for xanthine dehydrogenase, has been reported to be highly expressed in a lung adenocarcinoma (LUAD) subtype associated with poor survival [36]. In our analysis, XDH was hypomethylated in TSS200 and gene body, and was associated with up-regulated gene expression. We identified 9 candidate drugs targeting XDH. XDH inhibitors may be purine analogs e.g., allopurinol and oxypurinol, or non-purine agents, e.g., topiroxostat. The antitumor effects of allopurinol in NSCLC cell lines have been recently described, as well as a 6-gene signature for allopurinol-sensitive and allopurinol-resistant NSCLC cell lines [36]. Eniluracil, an orally active dihydropyrimidine dehydrogenase (DPD) inhibitor that enhances activity of chemotaxic agents, also emerged as a drug for XDH. Eniluracil has been shown to improve 5-fluorouracil (5-FU) efficacy by minimizing its side effects and/or making it orally available [41]. ATIC encodes a bifunctional protein that catalyzes the final 2 steps of de novo purine biosynthesis and has been reported to interact with ALK [37]. In this study, ATIC was hypomethylated in gene body and was associated with up-regulated gene expression. Of the 7 drugs found to target ATIC, pemetrexed is commonly used in NSCLC chemotherapy [42]. CA9 specifies a zinc-containing glycoprotein and has been implicated in tumorigenesis [38]. In the present study, it was identified to be hypermethylated in gene body but was related with up-regulated gene expression. Of the 6 drugs targeting CA9, benzthiazide [43], hydroflumethiazide [44], WX-G250 [45], and ellagic acid [46] have shown antitumor properties. SLC7A11 encodes the light chain subunit of cystine/glutamate antiporter system xc—and is involved in glutamine metabolism. This gene has been shown to modulate glucose and glutamine dependency in cancer cells [47]. In this analysis, SLC7A11 was hypomethylated in gene body and was related with up-regulated gene expression. Of the 5 drugs targeting SLC7A11, riluzole, a noncompetitive metabotropic glutamate receptor 1 (mGluR1) antagonist, and sulfasalazine, a cystine/glutamate antiporter system xc-inhibitor used to treat inflammatory bowel disease and arthritis, have antitumor properties [48,49,50]. Most recently, GAPDH has been identified as a potential prognostic biomarker or drug target of LUAD in a comprehensive proteomics analysis conducted by Jun-Yu Xu et al. [40]. In our study, GAPDH was hypomethylated in gene body and was associated with up-regulated gene expression, and also found as a drug interacting target. Of the 4 drugs targeting GAPDH, thionicotinamide-adenine-dinucleotide [51] have shown potent cytotoxicity against cancer cells.

### 3.8. Validation of Affinity of the Candidate Drugs by Molecular Docking Analysis

**Figure 9:**
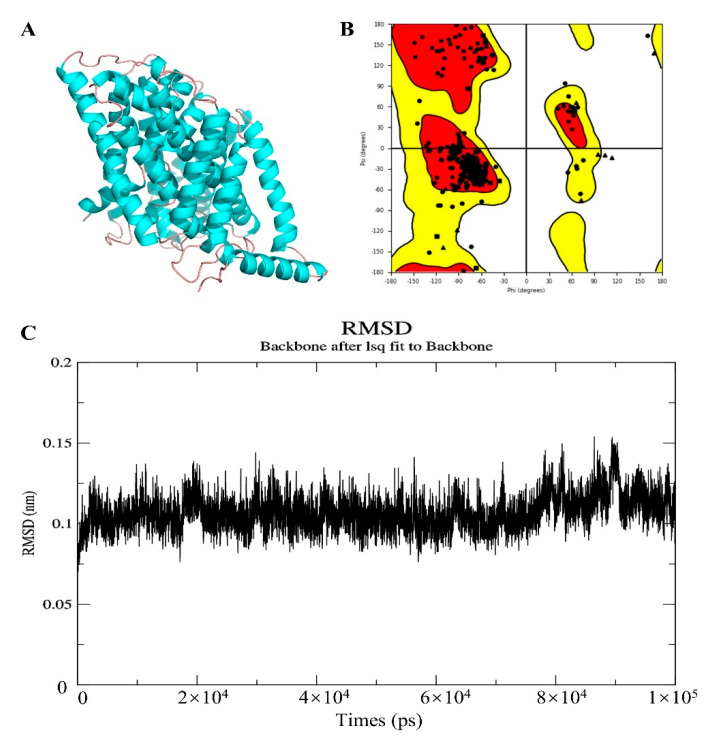
Homologous modeling of SLC7A11 protein structure. (**A**) 3D structure of SLC7A11. (**B**) Ramachandran plot analysis. (**C**) The root-mean-square deviation (RMSD) profile for time period of 100 ns.

**Figure 10:**
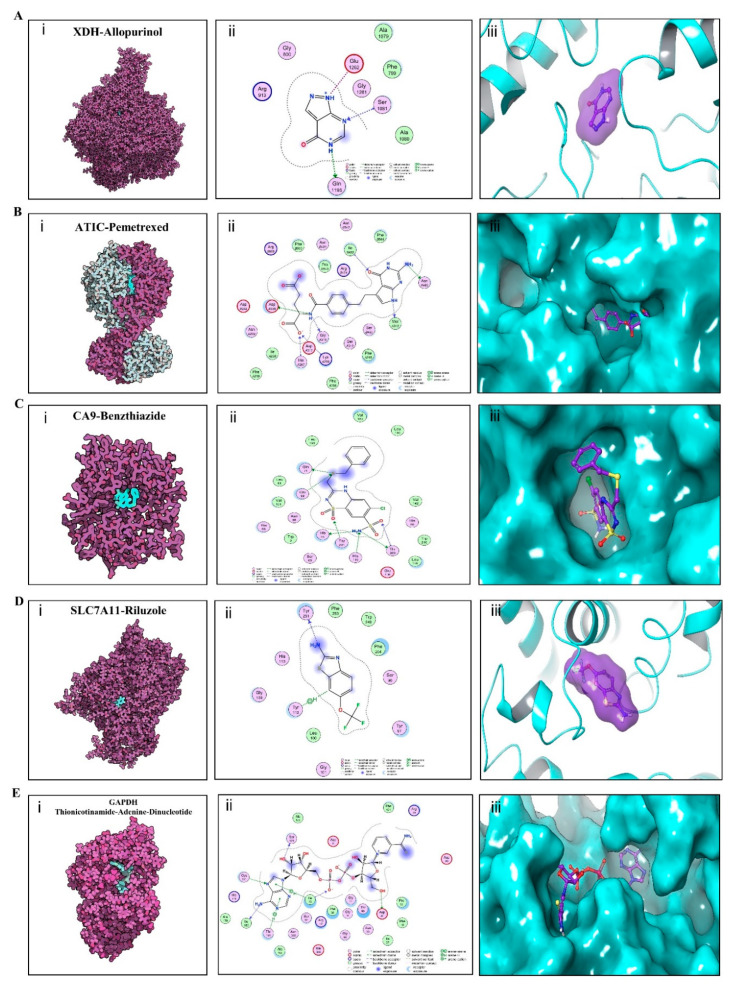
Binding mode of screened drugs to their targets by molecular docking. (**A**) Binding mode of XDH-Allopurinol complex. (**B**) Binding mode of ATIC-Pemetrexed complex. (**C**) Binding mode of CA9-Benzthiazide complex. (**D**) Binding mode of SLC7A11-Riluzole complex. (**E**) Binding mode of GAPDH-(Thionicotinamide-Adenine-Dinucleotide) complex. (**i**), Cartoon representation, overlay of the crystal structures of small molecule compounds and their targets were illustrated by Molecule of the Month feature. (**ii**), 2D interactions of compounds and their targets. (**iii**) 3D structures of binding interface were showed by PyMOL software.

To elucidate the binding mode of the candidate drugs for their targets, molecular docking analysis was performed. First, 3D model of SLC7A11 protein structure was predicted by the template-based homology modeling approach with SWISS-MODEL server. Consequently, large neutral amino acids transporter small subunit 1 (PDB ID: 6irt.1.B) was identified as ideal template for modeling as it indicated high sequence similarity (48.63%) (Figure 9A) [52]. Ramachandran plot analysis showed that 92.26% of the residues were present in the allowed area, demonstrating the accuracy of the predicted SLC7A11 structure (Figure 9B). The quality of the protein structure was further refined using molecular dynamics simulations method, and the stability of the protein model was estimated by root-mean-square deviation (RMSD) method. As shown in Figure 9C, the RMSD profile displayed the result of molecular dynamics of SLC7A11 model, identifying the final structure of SLC7A11 tended to be stable. The binding modes of targets and their drug candidates were analyzed by Autodock Vina v.1.1.2, and the binding energy for each target-drug interaction was generated (Figure 10 and Figure S3, Table 4). Results demonstrated that each drug candidate bound to its protein target primarily through strong electrostatic and hydrogen-bonding interactions. Furthermore, the active site of each target was occupied successfully by the candidate drugs. The binding energy for ATIC-Pemetrexed complex is −9.1 kcal/mol, and for GAPDH-Thionicotinamide-Adenine-Dinucleotide complex is −9.6 kcal/mol, indicating highly stable binding (Table 4).

## 4. Discussion

Patients with “EGFR Wild Type/Low PD-L1 expression” lung cancer lack a first-line single drug therapy as they hardly respond to TKIs and immune checkpoint inhibitors. Although response can be improved by combining anti PD-1 antibody therapy with conventional therapies, limitation of available drugs made it still a significant challenge for clinical practice to establish a fine balance between toxicity and therapeutic benefit [53]. Thus, novel therapies with less harmful side effects and better efficacy in combination are needed.

In this study, we performed an epigenome-genes association study of 133 patients from TCGA, which was validated in independent cohorts of patients with “EGFR Wild Type/Low PD-L1 expression” NSCLC from GEO. Compared with normal controls, “EGFR Wild Type/Low PD-L1 expression” NSCLC patients showed poor lymphocyte infiltration and downregulation of immune checkpoint proteins, meeting the criteria for classification as “cold” tumors [54,55]. Previous evidence has been found that DNA hypermethylation is related to immunity and immune response to ICIs [25,56]. Notably, the gene enrichment analysis for downregulated DMEGs involved in negatively regulating immune system process and T cell mediated immunity pathway, indicating DNA methylation also may act as a key role in maintaining the “cold” immune microenvironment.

Epigenetic changes have been associated with various cancers and DNA hypermethylation in CpG islands of tumor suppressor genes has been shown to inactivate them, thereby promoting cancer [57,58]. Similarly, we found that hypermethylated DMRs in 3 regions (TSS200, TSS1500, and gene body) are significantly higher than hypomethylated ones. Furthermore, 15 tumor suppressor genes belonging to the HyperDown group were identified, of which CDO1 (Cysteine Dioxygenase Type 1) [59,60], IRF8 (Interferon Regulatory Factor 8) [61], STAT5A (Signal Transducer And Activator Of Transcription 5A) [62], CFTR (CF Transmembrane Conductance Regulator) [63], ADAMTS8 (ADAM Metallopeptidase With Thrombospondin Type 1 Motif 8) [64], WIF1 (WNT Inhibitory Factor 1) [65], GATA5 (GATA Binding Protein 5) [66], FOXA2 (FOXA2) [67], SHISA3 (Shisa Family Member 3) [68], AXIN2 (Axin 2) [69], DIRAS3 (DIRAS family GTPase 3) [70], IRX1 (Iroquois Homeobox 1) [71], and ITGA5 (Integrin Subunit Alpha 5) [72] are confirmed by previous studies to be silenced via hypermethylation in lung cancer (Tables S1–S3). Although the tumor suppressor CAMK2N1 (Calcium/Calmodulin Dependent Protein Kinase II Inhibitor 1) has not been associated with lung cancer yet, its hypermethylation has been shown to promote tumorigenesis in other cancers [73]. These indicated that relative to other lung cancer types, “EGFR Wild Type/Low PD-L1 expression” NSCLC experiences more diversified epigenetic silencing of tumor suppressors, which made its carcinogenic mechanisms more complicated.

To evaluate the influence of genomic epigenetic changes on prognosis, we evaluated the prognostic power of DMEGs in “EGFR Wild Type/Low PD-L1 expression” NSCLC and a 4 DMEGs-based (PLCXD3, BAIAP2L2, NPR3, SNX10) prognostic model were identified using a LASSO regression analysis model. The biological roles of these four genes in “EGFR Wild Type/Low PD-L1 expression” NSCLCs have not been thoroughly investigated. PLCXD3 encodes a phospholipase that hydrolyzes phospholipids into fatty acids [74]. Its function in lung cancer is not clear yet. In our study, expression of PLCXD3 was negatively correlated with risk, indicating it can be identified as a protective factor. BAIAP2L2 (BAI1-associated protein 2-like 2) belongs to an I-BAR family and plays an important role in regulating membrane protrusions. Lei Xu et al. found BAIAP2L2 was upregulated in lung adenocarcinoma and acted as an oncogene in the development of lung cancer [75]. In line with their findings, expression of BAIAP2L2 was identified positively correlated with risk and negatively associated with OS. NPR3 (natriuretic peptide receptor 3) has been reported as one of the prognostic markers for colorectal cancer (CRC), for which upregulation signified poor survival [76]. In the present study, expression of NPR3 was positively correlated with risk and negatively associated with OS. SNX10 (sorting nexin 10) belongs to SNX family and contains a PX-domain. Several studies have revealed that SNX10 functioned as a tumor suppressor gene in progression of CRC [77,78]. Surprisingly, in our study, expression of SNX10 was positively correlated with risk and negatively associated with OS. This prognostic model effectively categorized training set samples into high- and low-risk classes and high area under the AUC effectively predicts 1-, 3-, and 5-year OS. KM analysis also revealed significant OS differences between the high- and low-risk. Taken together, these observations indicate that aberrant methylation significantly influences the pathogenesis of “EGFR Wild Type/Low PD-L1 expression” NSCLC, which was reflected in clinical prognosis.

Drug repurposing is a strategy for identifying new uses for approved or investigational drugs, which can significantly reduce the cost and time to bring a new treatment to patients [79,80]. We used DMEGs protein–drug interaction data to identify potential therapeutic candidates from DrugBank database. Remarkably, our analysis identified the drug target GAPDH, which has just been identified as a potential prognostic biomarker or drug target of LUAD in a comprehensive proteomics analysis on LUAD patients [40]. Besides, we identified pemetrexed, the only drug currently approved by the FDA for first-line use in combination with anti-PD1 antibodies against lung cancer regardless of PD-L1 expression [7,8,9], which indicated that our finding drugs may enrich the library of candidates for combination strategies based on immune checkpoint inhibitors. Riluzole, an SLC7A11 inhibitor used to manage ALS (Amyotrophic lateral sclerosis), and sulfasalazine, which is used to treat IBD (inflammatory bowel disease) and arthritis were also identified as potential candidates. Although not clinically used against cancer, both have been reported to have anticancer properties. Benzthiazide and hydroflumethiazide are used as diuretics in clinical practice and ellagic acid is also present in fruits, including strawberries and blueberries. Importantly, these candidates are known to be low toxicity. In this study, the binding modes of candidate drugs with the targets were further elucidated through docking analysis, offering a rational molecular explanation. Besides, the other treatment target rely on epigenetic signature elucidated which can be erasable by epigenetic drugs to enhance cold tumor response to immunotherapy [25]. Such drugs, including DNA demethylating agents [81,82] and deacetylase inhibitors, are in clinical use against some leukemias and lymphomas. Clinical trials of this class of drugs in combination with immune checkpoint inhibitors in lung cancer treatment are also ongoing [83].

## 5. Conclusions

In summary, the present study uncovered the distinct methylation-transcription characteristics of “EGFR Wild Type/Low PD-L1 expression” NSCLC, and provided an adaptable way to identify potential therapeutic targets, which may enrich the library of candidates for combination strategies based on immune checkpoint inhibitors against this intractable lung cancer subtype.

## Figures and Tables

**Table 1:** Differentially methylated and expressed genes (DMEGs) grouping standard.

Groups	Methylation Cut-Off	Expression Cut-Off
HypoUp	FDR < 0.01 and delta β-value < −0.2	FDR < 0.01 and log2FC > 1
HypoDown	FDR < 0.01 and delta β-value < −0.2	FDR < 0.01 and log2FC < −1
HyperUp	FDR < 0.01 and delta β-value > 0.2	FDR < 0.01 and log2FC > 1
HyperDown	FDR < 0.01 and delta β-value > 0.2	FDR < 0.01 and log2FC < −1

**Table 2:** 4-DMEGs based signature.

Symbol	Coef	HR	*p* Value	Low 95% CI	High 95% CI
BAIAP2L2	0.022	1.023	0.000	1.011	1.035
NPR3	0.011	1.011	0.042	1.000	1.023
PLCXD3	−0.102	0.903	0.222	0.768	1.063
SNX10	0.017	1.017	0.013	1.004	1.031

**Table 3:** Nine DMEGs targeted by available drugs.

RefGene	Region	Relation to Island	Pattern	Drugs	Drug Example
XDH	TSS200	OpenSea	HypoUp	9	Allopurinol, Eniluracil
	Body	OpenSea	HypoUp	9	Allopurinol, Eniluracil
ATIC	Body	S_Shore	HypoUp	7	Pemetrexed
CA9	Body	Island	HyperUp	6	Benzthiazide, Hydroflumethiazide, Ellagic Acid
SLC7A11	Body	OpenSea	HypoUp	5	Riluzole, Sulfasalazine
GAPDH	Body	S_Shore	HypoUp	4	Thionicotinamide-Adenine-Dinucleotide
PPIF	Body	S_Shore	HypoUp	4	Cyclosporine, L-Proline
AKR1B10	Body	OpenSea	HypoUp	3	Tolrestat
MMP11	Body	S_Shore	HypoUp	2	Marimastat
GMDS	Body	Island	HyperUp	2	Guanosine-5′-Diphosphate-Rhamnose, Guanosine-5′-Diphosphate

**Table 4:** Binding Energy for targets with their drugs.

Target	Drug	Binding Energy (kcal/mol)
XDH	Allopurinol	−6.0
XDH	Eniluracil	−5.8
CA9	Benzthiazide	−7.0
CA9	Ellagic-Acid	−7.1
CA9	Hydroflumethiazide	−6.2
ATIC	Pemetrexed	−9.1
GAPDH	Thionicotinamide-Adenine-Dinucleotide	−9.6
SLC7A11	Sulfasalazine	−9.8
SLC7A11	Riluzole	−7.2

## Data Availability

The datasets analyzed during the current study are available in the Genomic Data Commons (GDC, https://gdc-portal.nci.nih.gov/legacy-archive/) and Gene Expression Omnibus (GEO, https://www.ncbi.nlm.nih.gov/geo/) repositories.

## Supplementary Materials

The following are available online at https://www.mdpi.com/2072-6694/12/9/2496/s1, Figure S1: Venn mapping showing the intersection of DMEGs in TSS200, TSS1500 and gene body, Figure S2: The relationship between RiskScore and patient outcome in the validation cohort (from GEO), Figure S3: Binding mode of screened drugs to their targets by molecular docking, Table S1: The clinical characteristics of the study sample, Table S2: The regulated pattern of DMEGs in TSS200, Table S3: The regulated pattern of DMEGs in TSS1500, Table S4: The regulated pattern of DMEGs in gene body.

## Abbreviations

ICIs:	Immune checkpoint inhibitors;
NSCLC:	Non-small cell lung cancer;
EGFR:	Epidermal growth factor receptor;
PD-L1:	programmed death-ligand 1;
DMRs:	differentially methylated regions;
*PLCXD3*:	Phosphatidylinositol-Specific Phospholipase C, X Domain Containing 3;
*BAIAP2L2*:	BAR/IMD Domain Containing Adaptor Protein 2 Like 2;
*NPR3*:	Natriuretic Peptide Receptor 3;
*SNX10*:	Sorting Nexin 10;
*XDH*:	Xanthine Dehydrogenase;
*ATIC*:	5-Aminoimidazole-4-Carboxamide Ribonucleotide Formyltransferase/IMP Cyclohydrolase;
*CA9*:	Carbonic Anhydrase 9;
*SLC7A11*:	Solute Carrier Family 7 Member 11;
*GAPDH*:	Glyceraldehyde-3-Phosphate Dehydrogenase;
TKI:	Tyrosine kinase inhibitors;
PD1:	Programmed death 1;
*ALK*:	ALK receptor tyrosine kinase;
OS:	Overall survival;
IDO:	Indoleamine 2,3-dioxygenase;
VEGF:	Vascular endothelial growth factor;
CTLA-4:	Cytotoxic T-lymphocyte-associated protein 4;
LAG-3:	Lymphocyte-activation gene 3;
Tim-3:	T-cell immunoglobulin mucin 3;
TCGA:	The Cancer Genome Atlas;
DMEGs:	differentially methylated and expressed genes;
GEO:	Gene Expression Omnibus;
DMSs:	Differentially methylated CpG sites;
DMRs:	Differentially methylated regions;
DMGs:	Differentially methylated genes;
FDR:	False discovery rate;
BH:	Benjamini and Hochberg;
DEGs:	Differentially expressed genes;
GO:	Gene Ontology;
KEGG:	Kyoto Encyclopedia of Genes and Genomes;
BP:	Biological process;
CC:	Cellular component;
MF:	Molecular functions;
PCA:	Principal Component Analysis;
LOOCV:	Leave-one-out cross-validation;
ROC:	Receiver operating characteristic;
AUC:	Area under the curve;
LASSO:	Least Absolute Shrinkage and Selection Operator;
TIMER:	Tumor immune estimation resource;
ESTIMATE:	Estimation of STromal and Immune cells in MAlignant Tumor tissues using Expression data;
RMSD:	Root-mean-square deviation;
TSS	Transcription start sites
*CD274*:	CD274 molecule, also known as PD-L1;
*HAVCR2*:	Hepatitis A Virus Cellular Receptor 2, also known as TIM3;
*PDCD1*:	Programmed Cell Death 1, also known as PD1;
*PDCD1LG2*:	Programmed Cell Death 1 Ligand 2;
*CDO1*:	Cysteine Dioxygenase Type 1;
*IRF8*:	Interferon Regulatory Factor 8;
*STAT5A*:	Signal Transducer and Activator of Transcription 5A;
*CFTR:*	CF Transmembrane Conductance Regulator;
*ADAMTS8*:	ADAM Metallopeptidase with Thrombospondin Type 1 Motif 8;
*WIF1:*	WNT Inhibitory Factor 1;
*GATA5*:	GATA Binding Protein 5;
*FOXA2*:	FOXA2;
*SHISA3:*	Shisa Family Member 3;
*AXIN2*:	Axin 2;
*DIRAS3*:	DIRAS family GTPase 3;
*IRX1*:	Iroquois Homeobox 1;
*ITGA5*:	Integrin Subunit Alpha 5;
*CAMK2N1*:	Calcium/Calmodulin Dependent Protein Kinase II Inhibitor 1;
CRC:	Colorectal cancer;
SNX10:	Sorting nexin 10;
ALS:	Amyotrophic lateral sclerosis;
IBD:	Inflammatory bowel disease
