# Supplementary material for: Combined Methylome and Transcriptome Analyses Reveals Potential Therapeutic Targets for EGFR Wild Type Lung Cancers with Low PD-L1 Expression
Source: Cancers (Basel). 2020 Sep 3;12(9):2496. doi: 10.3390/cancers12092496 (PMC7563876; doi:10.3390/cancers12092496)
Supplement: Supplementary file 1 [file cancers-12-02496-s001.zip › cancers-903682 - final - supplementary material/cancers-903682 - supplementary material.pdf]

Supplementary Materials

Combined Methylome and Transcriptome Analyses Reveals Potential Therapeutic Targets for EGFR Wild Type Lung Cancers with Low PD-L1 Expression

Weilei Hu, Guosheng Wang, Lonny B. Yarmus and Yuan Wan

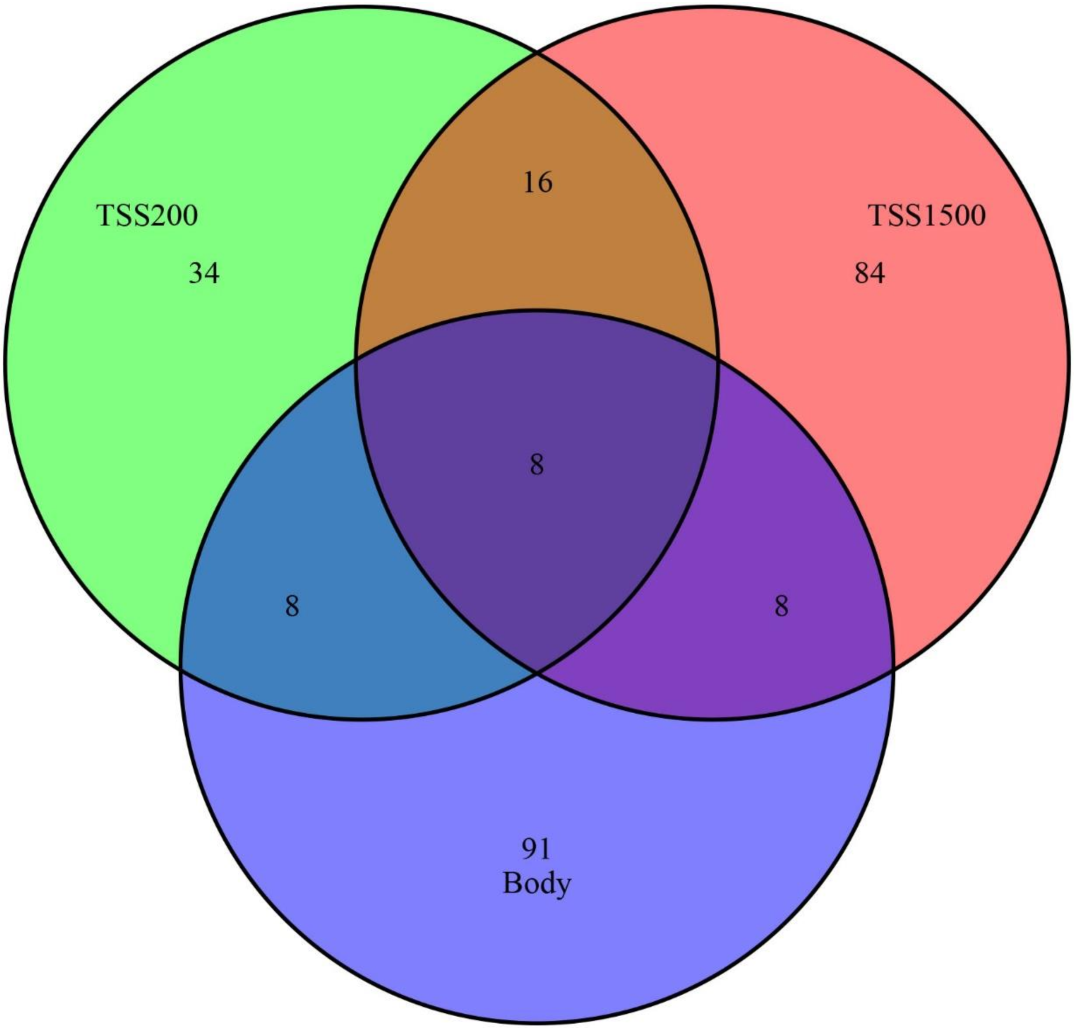

Figure S1. Venn mapping showing the intersection of DMEGs in TSS200, TSS1500 and gene body.

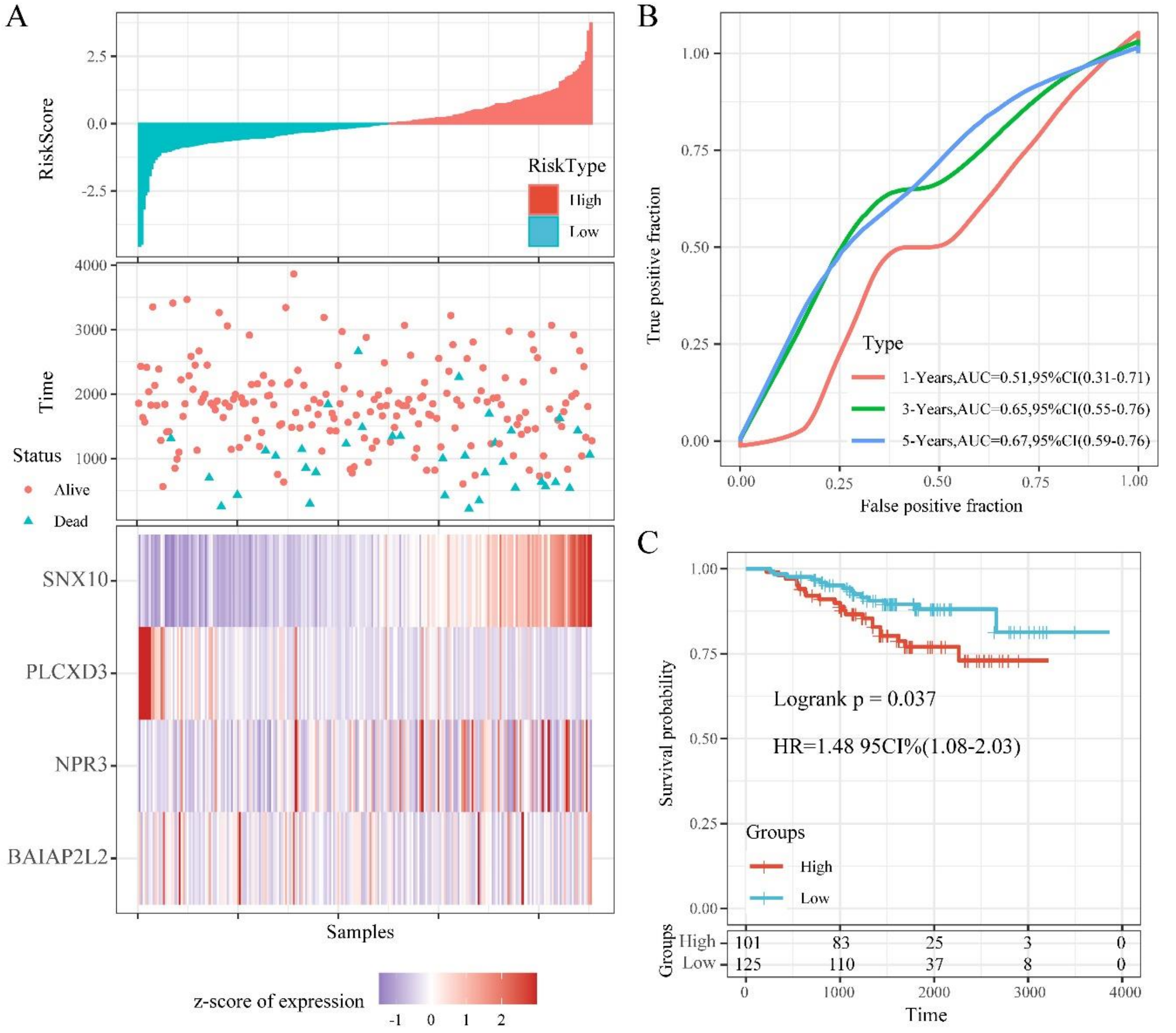

Figure S2. The relationship between RiskScore and patient outcome in the validation cohort (from GEO). (**A**) Each patient's RiskScore, survival time and status, and the expression of 4 DMEGs. The horizontal axis represents the samples, and the vertical axis represents RiskScores, OS, and immune-related gene expression, respectively. (**B**) 1-, 3-, and 5-years ROC analysis of prognosis classification for RiskScore. (**C**) KM survival analysis of patients with high RiskScore vs low RiskScore.

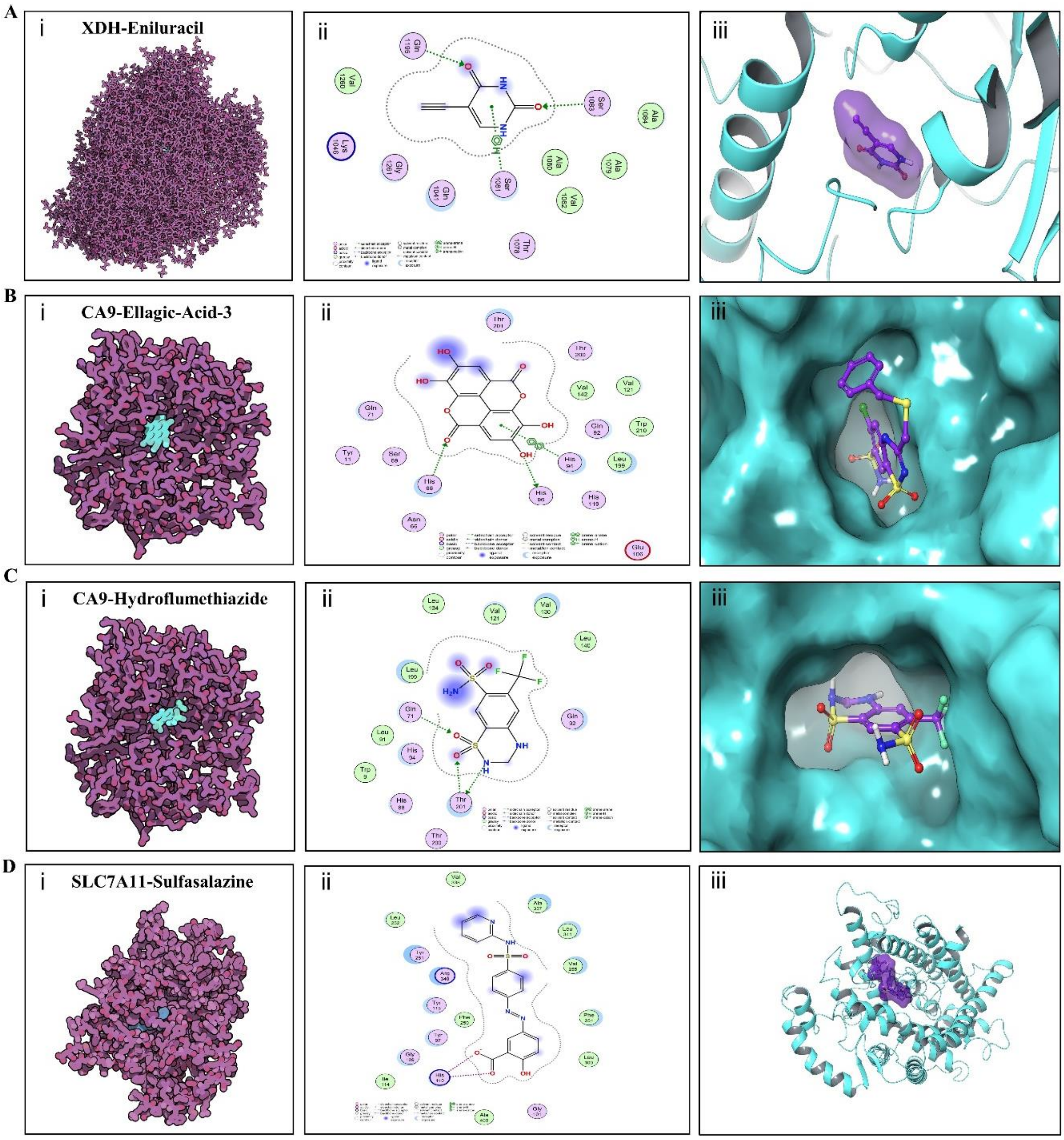

Figure S3. Binding mode of screened drugs to their targets by molecular docking. (**A**) Binding mode of XDH-Eniluracil complex. (**B**) Binding mode of CA9-(Ellagic-Acid-3) complex. (**C**) Binding mode of CA9-Hydroflumethiazide complex. (**D**) Binding mode of SLC7A11-Sulfasalazine complex. (**i**), Cartoon representation, overlay of the crystal structures of small molecule compounds and their targets were illustrated by Molecule of the Month feature. (**ii**), 2D interactions of compounds and their targets. (**iii**) 3D structures of binding interface were showed by PyMOL software.

© 2020 by the authors. Licensee MDPI, Basel, Switzerland. This article is an open access article distributed under the terms and conditions of the Creative Commons Attribution (CC BY) license (http://creativecommons.org/licenses/by/4.0/).
